# Supplementary material for: Ion rectification based on gel polymer electrolyte ionic diode
Source: Nat Commun. 2022 Nov 5;13:6669. doi: 10.1038/s41467-022-34429-9 (PMC9637189; doi:10.1038/s41467-022-34429-9)
Supplement: Supplementary file 1 — Supplementary Information [file 41467_2022_34429_MOESM1_ESM.pdf]

Supplementary Information for

Ion Rectification Based on Gel Polymer Electrolyte Ionic Diode

Fan Jiang[1], Wei Church Poh[1], Juntong Chen[1], Dace Gao[1], Feng Jiang[1], Xiaoyu Guo[1], Jian Chen[1], Pooi See Lee[1,*]

[1]School of Materials Science and Engineering

Nanyang Technological University 50 Nanyang Avenue, Singapore 639798,

Singapore

[*]Correspondence and requests for materials should be addressed to P.S.L.

(pslee@ntu.edu.sg)

1. Interfacial Toughness of PAZT/PHEC heterojunction

T-peel test was conducted to investigate the interfacial toughness of PAZT/PHEC heterojunction by using an MTS Criterion Model 42 electromechanical universal test system. T-peel tests were performed with a crosshead speed of 50 mm/min, according to the procedures in the ASTM D1876-08 standard. As shown in Supplementary Fig. 1(a), nylon filter (0.45 μm pore) and polycarbonate filter (0.2 μm pore) are employed as the substrates for tested GPEs (dimension 30 mm × 10 mm × 1 mm), which prevent GPEs' elongation along the peeling direction. It can be observed in Supplementary Fig. 1(b) that the soft PAZT GPE undergoes a cohesive failure near the interface during the T-peel test, leaving a residual layer on PHEC GPE. The failure within GPE indicates strong and durable adhesion at PAZT/PHEC interface, which is attributed to mutual solubility of GPEs due to having the same plasticizer (propylene carbonate). In Supplementary Fig. 1(c), the T-peel test of PAZT/PHEC heterojunction gives an adhesion energy of 38.00 ± 4.39 J/m^2 through 3 tested samples, comparable with the reported perfluorinated sulfonic acid (PFSA) GPEs [1]. Still, the cohesive failure occurs within the PAZT GPE instead of on the interface.

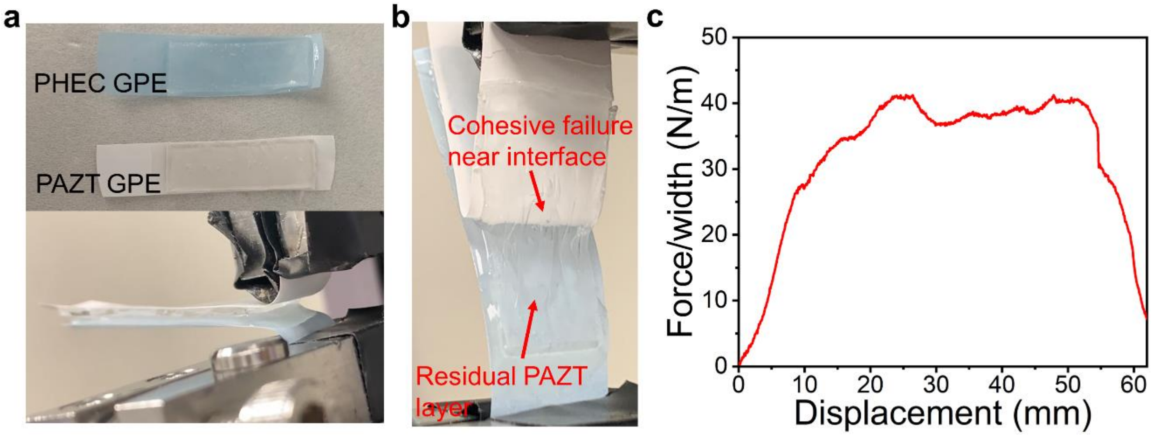

Supplementary Fig. 1 Strong adhesion of PHEC GPE with PAZT GPE. (a) image of a T-peel test setup; (b) Image of cohesive failure during a T-peel test of a PHEC GPE adhered with a PAZT GPE; (c) the force-displacement curves for a PHEC GPE bonded with a PAZT GPE.

2. Transient current response of fabricated junctions measured from − 2 V to + 2 V

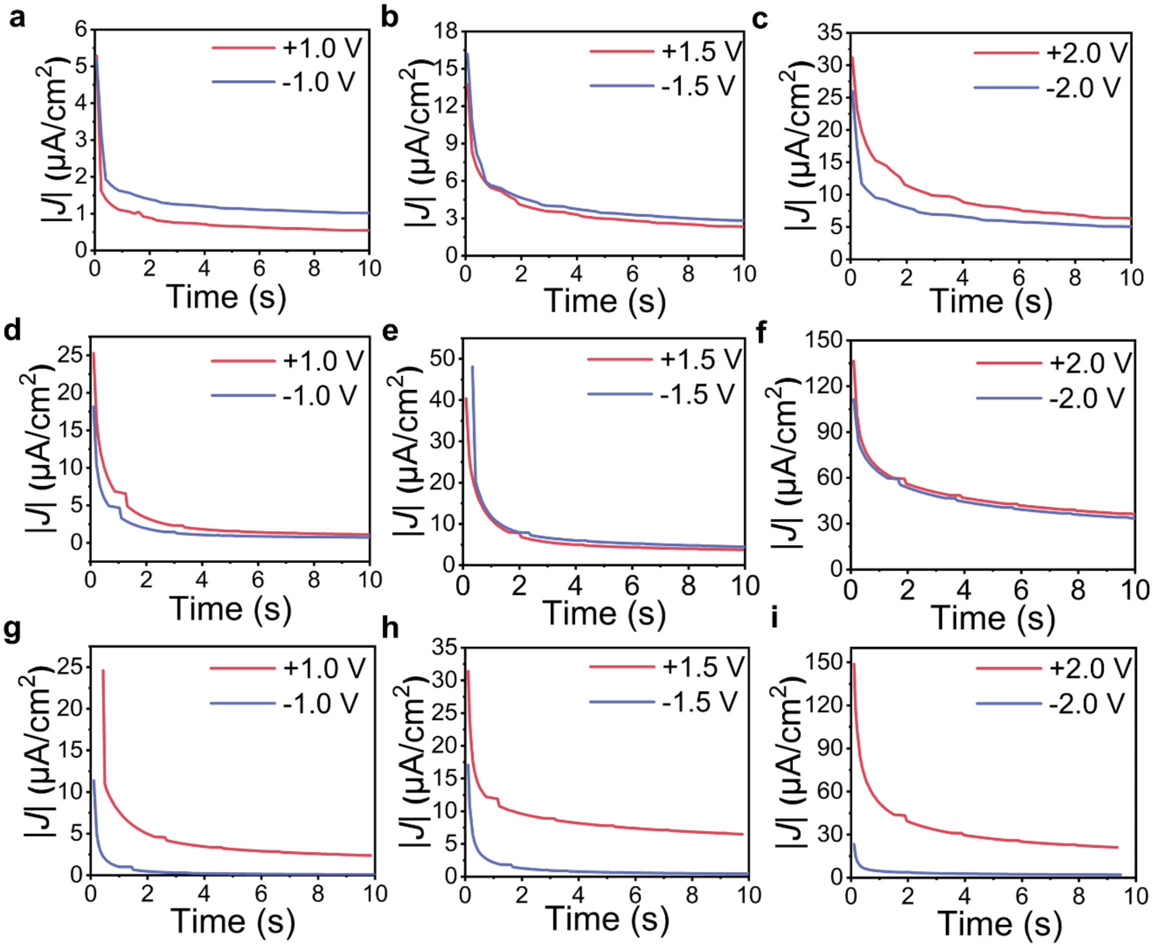

Supplementary Fig. 2 Transient current responses of GPE homojunctions and heterojunction. (a) PAZT/PAZT homojunction at ± 1 V bias; (b) PAZT/PAZT homojunction at ± 1.5 V bias; (c) PAZT/PAZT homojunction at ± 2.0 V bias; (d) PHEC/PHEC homojunction at ± 1.0 V bias; (e) PHEC/PHEC homojunction at ± 1.5 V bias; (f) PHEC/PHEC homojunction at ± 2.0 V bias; (g) PAZT/PHEC heterojunction at ± 1.0 V; (c) PAZT/PHEC heterojunction at ± 1.5 V; (i) PAZT/PHEC heterojunction at ± 2.0 V.

Supplementary Table 1 Measured current density of PAZT/PAZT, PHEC/PHEC and PAZT/PHEC under various voltage biases.

Sample	J under ± 1.0 V (μA/cm^2)		η (± 1.0 V)	J under ± 1.5 V (μA/cm^2)		η (± 1.5 V)	J under ± 2.0 V (μA/cm^2)		η (± 2.0 V)
	+1.0 V	−1.0 V		+1.5 V	−1.5 V		+2.0 V	−2.0 V	
PAZT/ PAZT	0.54	−1.01	0.54	2.28	−2.77	0.82	6.06	−4.97	1.22
PHEC/ PHEC	1.11	−0.83	1.34	3.74	−4.55	0.82	35.92	−33.52	1.07
PAZT/ PHEC	2.38	−0.103	23.11	6.50	−0.43	15.12	21.13	−2.07	10.21

The expressions of ion velocities which is employed to quantitatively describe ion migration as follow [2]:

$$v_+ - v_- = \frac{D_{0+}}{\mathrm{R}T}\frac{\mathrm{F}\Delta\emptyset}{L}, v_- - v_+ = -\frac{D_{0-}}{\mathrm{R}T}\frac{\mathrm{F}\Delta\emptyset}{L}$$

where v_+ and v_- are velocities of ions, D_{0+} and D_{0-} are diffusion coefficients of positive and negative ions, $\Delta\emptyset$ is the applied voltage, L is the thickness of GPE, R is gas constant, T is temperature and F is Faraday constant. According to the equation, ion velocities in polymer electrolytes are in linear relationship with ion diffusion coefficients and applied electric field. Thus, the connection between ion diffusion and migration can be established.

3. Electrical Stability and Durability of Ionic Diode

To test the stability of ion rectification in GPE ionic diode, a prolonged period of ± 1.0 V is applied. As shown in Supplementary Fig. 3(a), the current-time curves produced by the ± 1.0 V remains to be smooth and stable in the 200 s measurement, indicating good durability for long-term operation. The rectifying ratio (η) reaches high value of 24.0 at 3.5 s and starts to decrease at 12.6 s. The rectifying ratio decays to 80% (η = 19.2) at 29.5 s and 50% (η = 12.0) at 114.4 s, finally 41.4% (η = 9.95) at the 200 s. Moreover, the durability of the GPE ionic diode under a square-wave voltage of ± 1 V with 0.1 Hz frequency was also measured and displayed in Supplementary Fig. 3(b). The rectifying ratio of GPE ionic

diode decreased to 75.8% of initial performance after 50 cycles, then 56.2 % of initial performance remains after 100 cycles of alternating voltage.

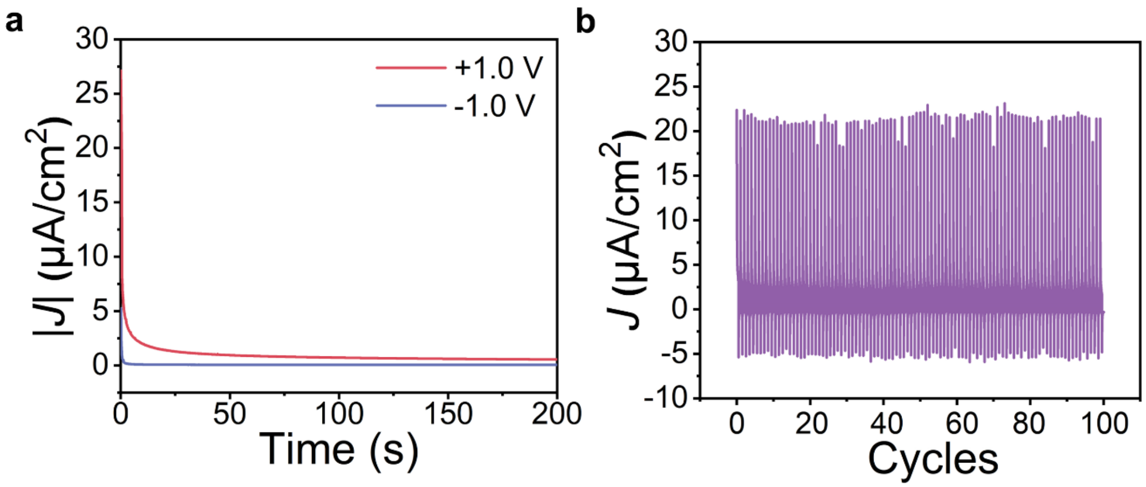

Supplementary Fig. 3 Electrical stability and durability of GPE ionic diode. (a) Transient current response of PAZT/PHEC heterojunction at ± 1.0 V for 200 s; (b) performance stability of the PAZT/PHEC diode a square-wave voltage of ± 1.0 V with 0.1 Hz frequency.

4. Chemical Analysis through XPS

XPS was employed to characterize the chemical bonds in the GPE ionic diode which has been applied with voltage at − 2 V and 2 V for 10 s, in order to confirm the chemical compositions and investigate possible chemical reactions. As seen in Supplementary Fig. 4(a), the PAZT GPE is composed of Zn, S, C, O and F elements. It could be observed in Supplementary Fig. 4(b) that two peaks Zn 2$p_{3/2}$ and Zn 2$p_{1/2}$ centered at 1022.3 eV and 1045.6 eV with energy width ~ 23 eV, suggesting the ionic state of zinc elements (Zn^{2+}) [3]. In Supplementary Fig. 4(c), the separated S 2*p* peaks revealed the oxidation state of sulfur elements, combined with the separated peak of O 1*s* (Supplementary Fig. 4(d)) at 533.6 eV (S-O/S=O), the chemical condition of sulfur at triflate salt could be confirmed [4, 5]. The peak centered at 688.4 eV in F 1*s* plot (Supplementary Fig. 4(e)) indicated the C-F bonds in the $CF_3O_3S^-$.

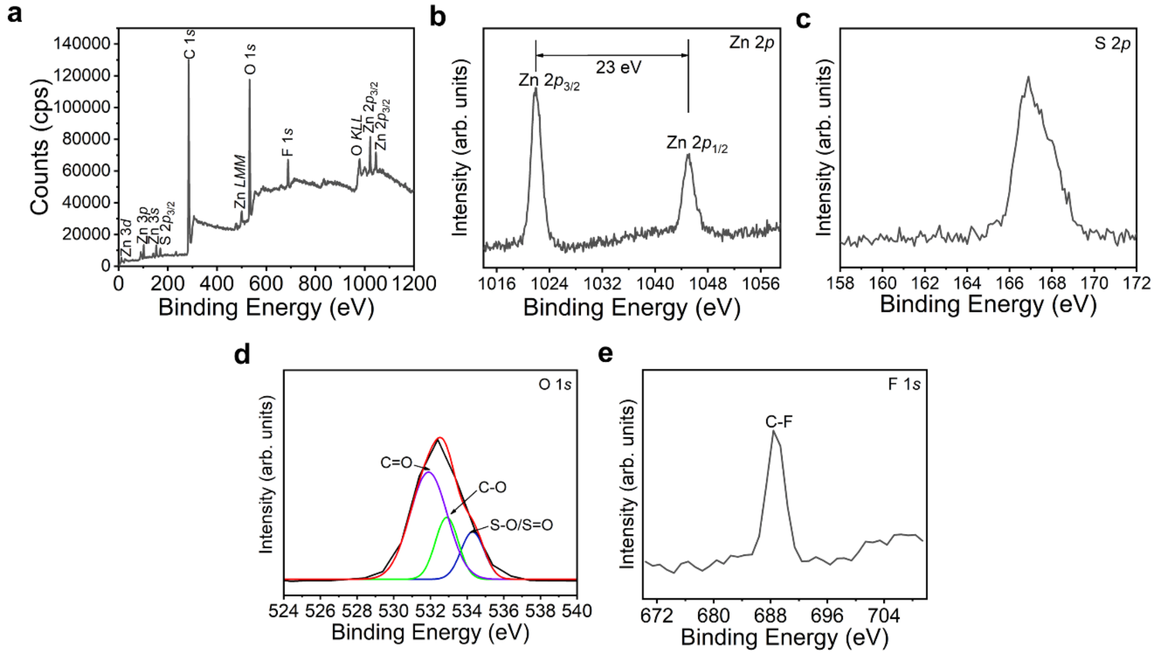

Supplementary Fig. 4 XPS spectra of PAZT from ionic diode. (a) full spectrum; (b) S 2*p*; (c) S 2*p*; (d) O 1*s*; (e) F 1*s*.

As presented in Supplementary Fig. 5(a), Cl, N, C, O and F elements existed in the PHEC part of ionic diode. The F 1*s* peak centered at 686.4 eV in Supplementary Fig 5(b) corresponded with the CF_3 group in PVDF-HFP. In Supplementary Fig. 5(c), the spin-orbit splitting of Cl 2*p* with energy width of 1.6 eV could be observed. The chloride ions were then identified by the Cl $2p_{3/2}$ peak centered at 195.7 eV. C-N and C=N bonds could be recognized at 287.1 and 285.8 eV in C 1*s* spectrum (Supplementary Fig. 5(d)), as well as separated peaks at 399.7 and 400.5 eV in N 1*s* spectrum (Supplementary Fig. 5(e)) respectively, which are derived from $[EMIM]^+$ ions [6, 7].

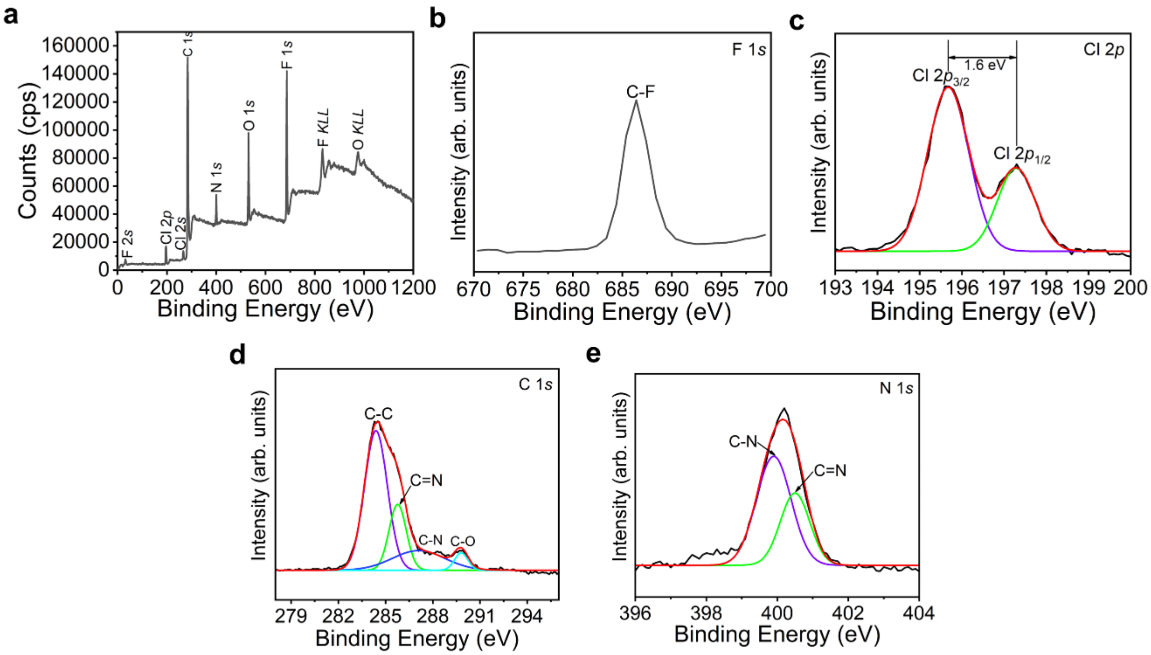

Supplementary Fig. 5 XPS spectra of PHEC from ionic diode. (a) full spectrum; (b) F 1*s*; (c) Cl 2*p*; (d) C 1*s*; (e) N 1*s*.

5. Electrochemical Properties of the Ionic Diode

The cyclic voltammograms measured with various scan rates of PHEC/PHEC homojunction and PAZT/PAZT homojunction are plotted in Supplementary Fig. 6(a) and (b), respectively. Owing to good stability and wide electrochemical window of PC solvent and ionic liquids, no redox reaction peak or electrochemical reaction could be observed in the two homojunction. In addition, the magnitude of current density obtained in both CV curves is below the limit of cut-off current density (0.1 mA/cm^2) selected to identify electrochemical window [8]. Therefore, only non-faradaic ion diffusion process happens within the gel polymer electrolytes. Both PAZT and PHEC gel polymer electrolytes could be regarded as electrochemically stable systems that could stand voltage bias up to 2 V. And the sharp CV curves suggest the low capacitance of the polymer/IL/FTO glass system, which is a good indication that charges are not accumulated at the GPE/electrode interface. As shown in Supplementary Fig. 6(c), no redox peaks could be observed for the PAZT/PHEC heterojunction ionic diode. In addition, the asymmetric curves in the heterojunction PAZT/PHEC with higher slope under positive voltage starting from 0 V also indicated faster ion diffusion for $[EMIM]^+$ and $CF_3O_3S^-$ ions.

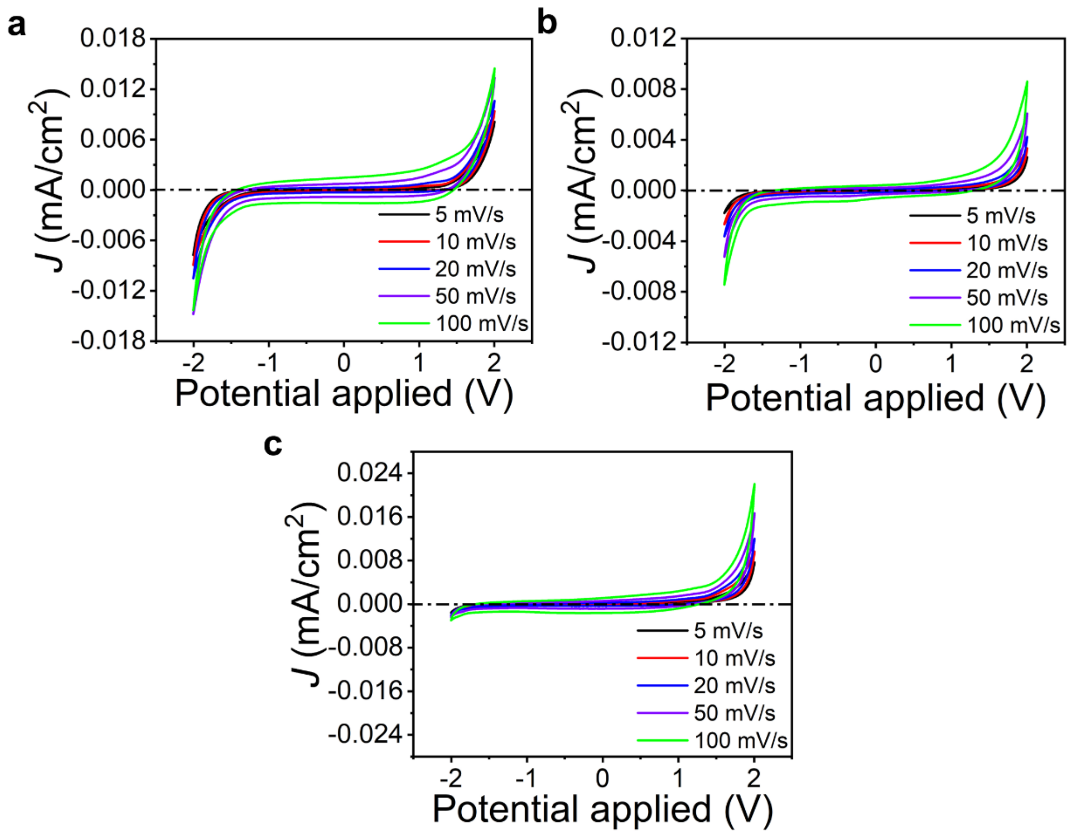

Supplementary Fig. 6 CV plots for homojunction and heterojunction at various scan rates. (a) PHEC/PHEC scanned from – 2 V to + 2 V; (b) PAZT/PAZT scanned from – 2 to + 2 V; (c) PAZT/PHEC scanned from – 2 V to + 2 V.

6. Simulation and fitting of EIS plots

The obtained CPE (constant phase element) describes the performance of a double layer, which could be used to calculate the capacitance of IDL with the equation: $C = Q^{1/n} R_{\mathrm{B}}{}^{(1/n-1)}$ [9].

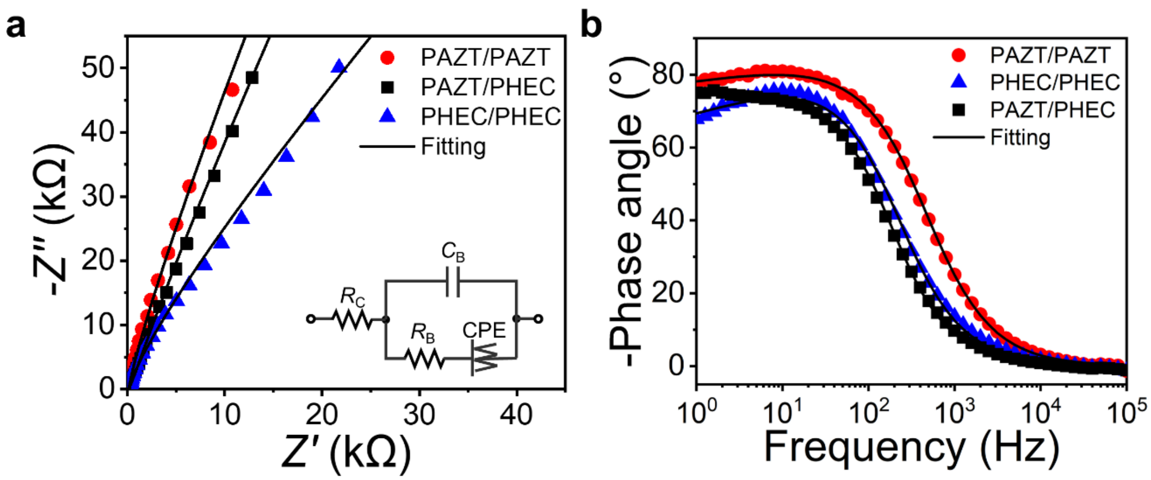

Supplementary Fig. 7 EIS tests of GPE ionic diode. (a) Nyquist plots for PHEC/PHEC, PAZT/PAZT and PHEC/PAZT; (b) Bode phase plots of PHEC/PHEC, PAZT/PAZT and PHEC/PAZT.

Supplementary Table 2 The EIS fitting parameters of PAZT/PHEC, PAZT/PAZT and PHEC/PHEC.

	R_C (Ω)	R_B (Ω)	CPE_{IDL}		C_B (μF)
			Q_{IDL}	n	
PAZT/PAZT	286.8 ± 6.2	331.7 ± 8.0	$(3.44 \pm 0.13)\times10^{-6}$	0.73 ± 0.02	2.14 ± 0.03
PHEC/PHEC	193.1 ± 3.4	252.6 ± 19.9	$(9.16 \pm 0.50)\times10^{-6}$	0.62 ± 0.02	3.04 ± 0.38
PAZT/PHEC	241.3 ± 7.5	2610.0 ± 115.3	$(7.45 \pm 0.28)\times10^{-6}$	0.70 ± 0.03	2.85 ± 0.25

7. Elemental Analysis through EDS

To further prove the formation of IDL and the diffusion-migration-based rectifying effect, elemental analysis through SEM-EDS is adopted. According to the proposed ionic diode model (Fig. 1), Cl^- and Zn^{2+} ions would accumulate and block at the interface due to the entropy-driven diffusion and the low diffusion rate in the counterpart GPE; while $[EMIM]^+$ and $CF_3O_3S^-$ ions would be driven away from the interface by the formed IDL. In the whole polymer/solvent/electrolyte system, only $[EMIM]^+$ ($(C_6H_{11}N_2)^+$) contain nitrogen and only $CF_3O_3S^-$ have sulfur. Thus, by detecting the distribution and analyzing the concentration of Zn, S, Cl and N elements around the interface of PAZT/PHEC

heterojunction, we expect to verify the principle of this rectifying effects through elemental analyses.

In Supplementary Fig. 8, the measured SEM images and EDS spectra of PAZT/PHEC heterojunction are presented. The Supplementary Fig. 8(a) and Supplementary Fig. 8(b) correspond with the PHEC and PAZT part near the interface, respectively; while Supplementary Fig. 8(c) and Supplementary Fig. 8(d) correspond with the middle part of PHEC and PAZT, respectively. As shown in Supplementary Fig. 8(b) and (d), the smooth and even surface morphology of PAZT derives from the amorphous characteristic of PMMA polymer; and the porous structures of PHEC originate from the fast evaporation of acetone and quick formation of PVDF-HFP polymer matrix.

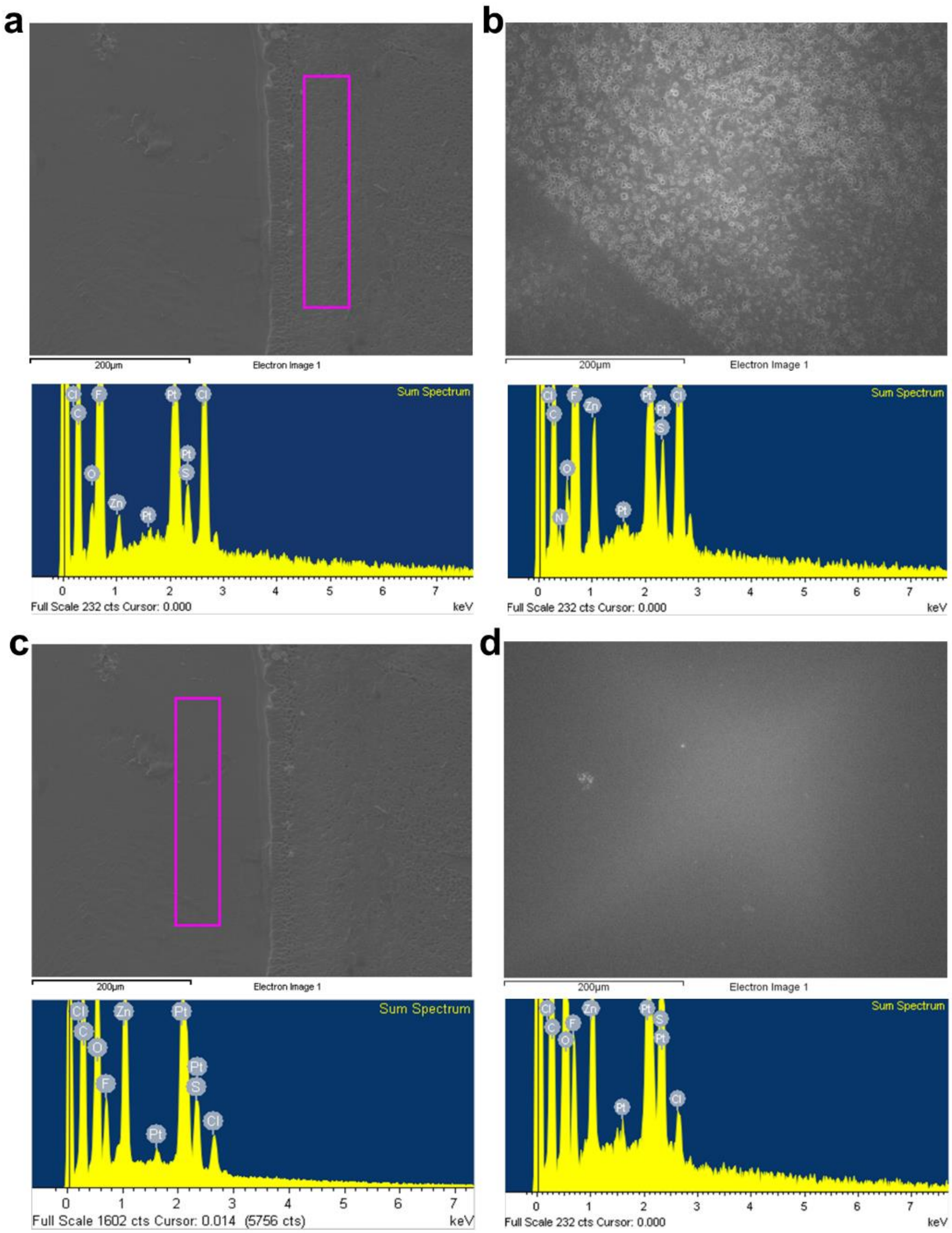

Supplementary Fig. 8 SEM images and EDS spectra of PAZT/PHEC heterojunction. (a) near interface at the PHEC part; (b) bulk area at the PHEC part; (c) near interface at the PAZT part; (d) bulk area at the PAZT part.

In Supplementary Table 3, the atomic concentrations of the 4 key elements distributed in the PAZT/PHEC heterojunction are organized and summarized. Near the interface of heterojunction (Supplementary Fig. 8(a)), the concentration of Cl in PHEC reaches 2.57 at% while N element ($[EMIM]^+$) cannot be detected. Then in the PHEC part (Supplementary Fig. 8(c)), the atomic ratio of N/Cl reaches 1.98, which fits the stoichiometric ratio of [EMIM]Cl (N : Cl = 2 : 1). As shown in Supplementary Fig. 8(d) for PAZT at bulk area, the ratio of S (3.72 at%) to Zn (1.75 at%) is 2.13, which corresponds well with the stoichiometric ratio in doped salt $Zn(CF_3O_3S)_2$ (S : Zn= 2 : 1). However, higher amount of Zn (4.20 at%) and lower amount of S (2.82 at%) were measured at the interface of PAZT (Supplementary Fig. 8(b)). The calculated S/Zn ratio of 0.67 indicated accumulation of Zn^{2+} ions around the interface.

Therefore, the test result fits our hypothesis model. Cl^- and Zn^{2+} ions would accumulate at the interface of PAZT/PHEC, and a small amount of Cl^- and Zn^{2+} ions could manage to trespass the interface to the counterpart GPE. Meanwhile, $[EMIM]^+$ and $CF_3O_3S^-$ ions would be pushed away and can be better detected far away from interface.

Supplementary Table 3 Atomic concentration of elements in the PAZT/PHEC ionic diode.

	N (Atomic%)	Cl (Atomic%)	N/Cl (Atomic%)	S (Atomic%)	Zn (Atomic%)	S/Zn (Atomic%)
PAZT-interface	–	1.68	–	2.82	4.20	0.67
PHEC-interface	–	2.57	–	0.87	1.15	0.76
PAZT-bulk	–	0.52	–	3.72	1.75	2.13
PHEC-bulk	6.42	3.24	1.98	1.09	1.63	0.67

8. Principle of Selecting Materials and Method

Gel polymer electrolyte (GPE) is composed of polymer matrix, solvent and ions. Due to the transparency, thermal stability and flexibility, PMMA/PC system was chosen. To facilitate an ionic diode, PVDF-HFP/PC was selected as another GPE due to the good plasticity and mechanical strength.

In the proposed ionic diode, the negative ions in the PMMA GPE and positive ions in the PVDF-HFP GPE could diffuse and migrate freely in the whole diode; while positive ions in the PMMA GPE and negative ions in the PVDF-HFP GPE would fail to diffuse through the interface and enter the counterpart GPE due to the low migration rates. Screening of cations and anions has been conducted through a series of salt solubility tests in the polymer matrix containing the selected salts and ionic liquids (shown in Supplementary Table 4 and 5). To satisfy the conditions above, [EMIM]Cl was picked as the ionic liquid for PVDF-HFP GPE, $Zn(CF_3O_3S)_2$ was chosen as the added ion source for PMMA GPE.

Supplementary Table 4 Solubility of salts in PMMA and PVDF-HFP polymer matrix

Salts	Solubility in PMMA	Solubility in PVDF-HFP	Reference
[EMIM][TFSI]	Soluble	Soluble	[10], solubility tests
Li[TFSI]	Soluble	Soluble	[11], solubility tests
$[EMIM]CF_3O_3S$	Soluble	Soluble	[12, 13], solubility tests
[EMIM]Cl	Insoluble	Soluble	[14], solubility tests
$Zn(CF_3O_3S)_2$	Soluble	Insoluble	[15], solubility tests

Supplementary Table 5 Solubility of chosen ions in PMMA and PVDF-HFP polymer matrix

	Solubility in PMMA	Solubility in PVDF-HFP
$[EMIM]^+$	Soluble	Soluble
Cl^-	Insoluble	Soluble
$CF_3O_3S^-$	Soluble	Soluble
Zn^{2+}	Soluble	Insoluble

9. Comparison of diffusion coefficients through EIS

Due to the linear correlation between differential capacitance (C_{Diff}) and diffusion coefficients of doped salts [16], the ion-diffusion-based mechanism could be further proved by comparing the capacitance derived from EIS. The total electrical impedance is given by the equation:

$$Z = Z_{\mathrm{RE}} + jZ_{\mathrm{Im}} = R + (jC\omega)^{-1} \quad (1)$$

$$-Z_{\mathrm{Im}} = (C\omega)^{-1} = (2\pi Cf)^{-1} \quad (2)$$

where Z_{Re} and Z_{Im} are the real and imaginary parts, and ω stands for angular frequency of the input ac perturbation [17]. Thus, the differential capacitance of the system could be calculated by measuring slope of impedance spectrum at low frequency region [18]. Here we doped both PMMA and PVDF-HFP gel polymer electrolytes with different salts in the same concentration of 0.2 mmol/g. By measuring the slope of bode plot shown in Supplementary Fig. 9 and employed the equation (2), we calculated and listed the differential capacitances of PMMA and PVDF-HFP GPEs in Supplementary Table 6. The C_{Diff} value for PMMA/$ZnCl_2$ is 5.48 μF, roughly one half as large as the 11.92 μF of PMMA/$Zn(CF_3O_3S)_2$, indicating higher ion diffusion coefficient of $CF_3O_3S^-$ ions than Cl^- in PMMA GPE. Similarly, the differential capacitance of PVDF-HFP/[EMIM]Cl is 14.01 μF, which is more than 2 times larger than value of PVDF-HFP/$ZnCl_2$, suggesting higher ion diffusion of $[EMIM]^+$ ions in PVDF-HFP GPE and validity of the mechanism.

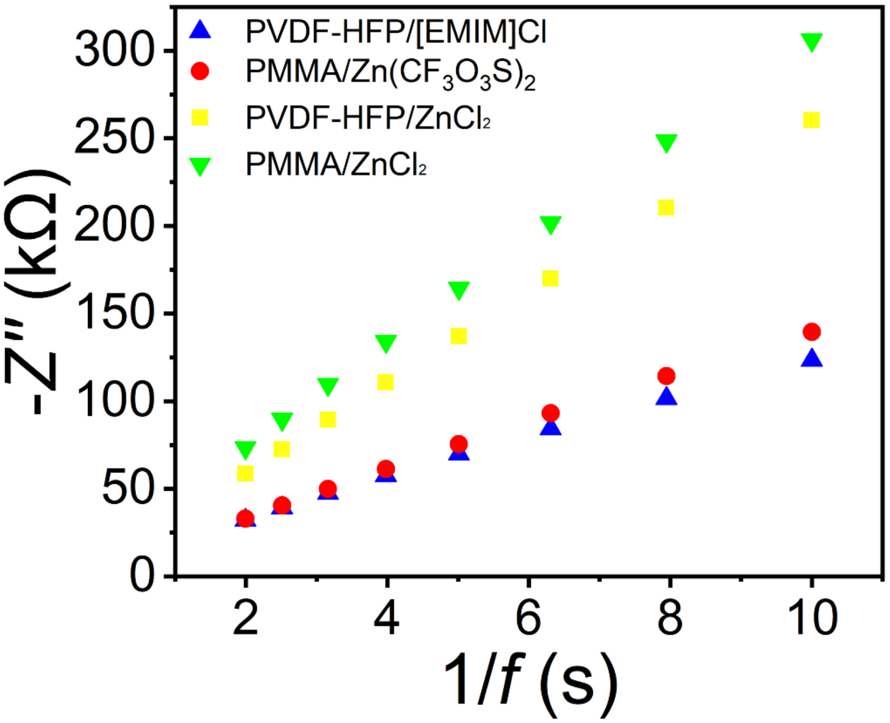

Supplementary Fig. 9 Measured Bode plot of PMMA and PVDF-HFP GPE with different salts.

Supplementary Table 6 Differential capacitances of PMMA and PVDF-HFP gel polymer electrolytes with different salts.

Salt \ Polymer	Capacitance of PMMA GPE (μF)	Capacitance of PVDF-HFP GPE (μF)
$ZnCl_2$	5.48	6.32
$Zn(CF_3O_3S)_2$	11.92	-
[EMIM]Cl	-	14.01

Supplementary References

[1] Oh, K. H., Kang, H. S., Choo, M. J., Jang, D. H., Lee, D., Lee, D. G., Kim, T. H., Hong, Y. T., Park, J. K., Kim, H. T. Interlocking Membrane/Catalyst Layer Interface for High Mechanical Robustness of Hydrocarbon-Membrane-Based Polymer Electrolyte Membrane Fuel Cells. *Advanced Materials* **27**, 2974-80 (2015).

[2] Choo, Y., Halat, D. M., Villaluenga, I., Timachova, K., & Balsara, N. P. Diffusion and migration in polymer electrolytes. *Progress in Polymer Science* **103**, 101220 (2020).

[3] Zhu, K., Wu, T. and Huang, K. A High Capacity Bilayer Cathode for Aqueous Zn-Ion Batteries. *ACS Nano* **13**, 14447–14458 (2019).

[4] Hartmann, P. et al. A comprehensive study on the cell chemistry of the sodium superoxide (NaO_2) battery. *Phys. Chem. Chem. Phys.* **15**, 11661–72 (2013).

[5] Liu, B. et al. Enhanced Cyclability of Lithium–Oxygen Batteries with Electrodes Protected by Surface Films Induced via In Situ Electrochemical Process. *Advanced Energy Materials* **8**, 1702340 (2018).

[6] Foelske-Schmitz, A. & Sauer, M. About charging and referencing of core level data obtained from X-ray photoelectron spectroscopy analysis of the ionic liquid/ultrahigh vacuum interface. *Journal of Electron Spectroscopy and Related Phenomena* **224**, 51–58 (2018).

[7] Yan, X. et al. Preparation and characterization of electrochemically deposited carbon nitride films on silicon substrate. *Journal of Physics D: Applied Physics* **37**, 907–913 (2004).

[8] Hayyan, M. et al. Investigating the electrochemical windows of ionic liquids. *Journal of Industrial and Engineering Chemistry* **19**, 106–112 (2013).

[9] Kim, H. J., Chen, B., Suo, Z. et al. Ionoelastomer junctions between polymer networks of fixed anions and cations. *Science* **367**, 773–776 (2020).

[10] Kim, Y. M. et al. A facile random copolymer strategy to achieve highly conductive polymer gel electrolytes for electrochemical applications. *Journal of Materials Chemistry C* **7**, 161–169 (2019).

[11] Ye, H. et al. Li Ion Conducting Polymer Gel Electrolytes Based on Ionic Liquid/PVDF-HFP Blends. *J. Electrochem. Soc.* **154**, A1048–A1057 (2007).

[12] Lewandowski, A. and Świderska, A. Solvent-free double-layer capacitors with polymer electrolytes based on 1-ethyl-3-methyl- imidazolium triflate ionic liquid. *Applied Physics A* **82**, 579–584 (2005).

[13] Liu, J. et al. Ionic Liquid-Incorporated Zn-Ion Conducting Polymer Electrolyte Membranes. *Polymers (Basel)* **12**, 1755 (2020).

[14] Correia, D. M. et al. Effect of Ionic Liquid Content on the Crystallization Kinetics and Morphology of Semicrystalline Poly(vinylidene Fluoride)/Ionic Liquid Blends. *Crystal Growth & Design* **20**, 4967–4979 (2020).

[15] Girish Kumar, G. & Sampath, S. Electrochemical and spectroscopic investigations of a gel polymer electrolyte of poly(methylmethacrylate) and zinc triflate. *Solid State Ionics* **176**, 773–780 (2005).

[16] Macounová, K. et al., Electrochemical Behavior of Nanocrystalline $Ru_{0.8}Me_{0.2}O_{2-x}$ (Me=Fe, Co, Ni) Oxide Electrodes in Double-Layer Region. *Journal of The Electrochemical Society* **154**, A1077 (2007).

[17] Lockett, V. et al. Differential Capacitance of the Electrical Double Layer in Imidazolium-Based Ionic Liquids: Influence of Potential, Cation Size, and Temperature. *The Journal of Physical Chemistry C* **112**, 7486–7495 (2008).

[18] Lockett, V. et al. Differential capacitance of the double layer at the electrode/ionic liquids interface. *Phys Chem Chem Phys* **12**, 12499–512 (2010).
